# Electron tomography of mouse LINC complexes at meiotic telomere attachment sites with and without microtubules

**DOI:** 10.1038/s42003-019-0621-1

**Published:** 2019-10-14

**Authors:** Marie-Christin Spindler, Josef Redolfi, Frederik Helmprobst, Philip Kollmannsberger, Christian Stigloher, Ricardo Benavente

**Affiliations:** 1 Department of Cell and Developmental Biology, Biocenter, University of Wuerzburg, 97074 Wuerzburg, Germany; 2 Imaging Core Facility, Biocenter, University of Wuerzburg, 97074 Wuerzburg, Germany; 3 Center for Computational and Theoretical Biology, University of Wuerzburg, 97074 Wuerzburg, Germany

**Keywords:** Electron microscopy, Nuclear envelope, Spermatogenesis, Telomeres

## Abstract

Telomere movements during meiotic prophase I facilitate synapsis and recombination of homologous chromosomes. Hereby, chromosome movements depend on the dynamic attachment of meiotic telomeres to the nuclear envelope and generation of forces that actively move the telomeres. In most eukaryotes, forces that move telomeres are generated in the cytoplasm by microtubule-associated motor proteins and transduced into the nucleus through the LINC complexes of the nuclear envelope. Meiotic LINC complexes, in mouse comprised of SUN1/2 and KASH5, selectively localize to the attachment sites of meiotic telomeres. For a better understanding of meiotic telomere dynamics, here we provide quantitative information of telomere attachment sites that we have generated with the aid of electron microscope tomography (EM tomography). Our data on the number, length, width, distribution and relation with microtubules of the reconstructed structures indicate that an average number of 76 LINC complexes would be required to move a telomere attachment site.

## Abstract (web summary)

Marie-Christin Spindler et al. present quantitative information on telomere attachment to LINC complexes during meiosis using electron microscope tomography. They show that an average of 76 LINC complexes is needed to move a telomere attachment site.

## Introduction

**Fig. 1:**
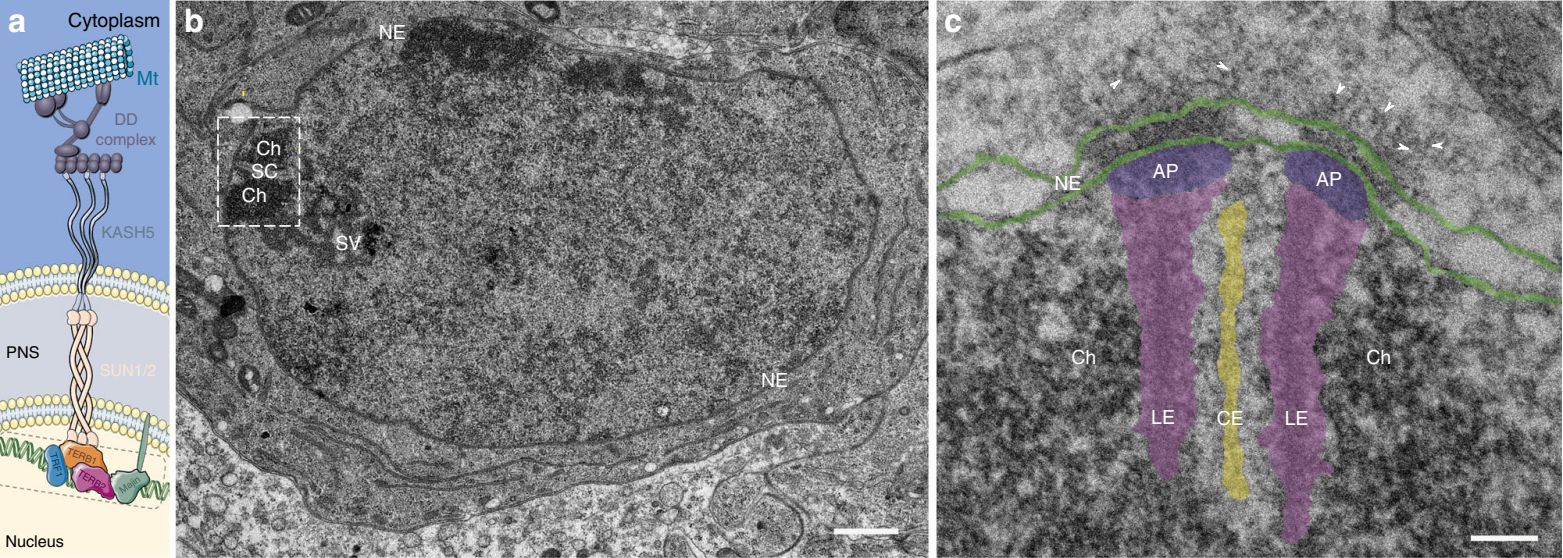
Transmission electron micrographs of murine telomere attachment sites to the nuclear envelope. **a** Schematic depiction of the molecular composition of meiotic telomere attachment sites. Telomere chromatin, which is bound by the meiotic telomere proteins (TRF1, TERB1/2, and Majin^46^), that are likely part of the attachment plate. LINC complexes anchored in the inner nuclear membrane that consist of the hetero-hexameric complex of inner nuclear membrane protein SUN1/2 and outer nuclear membrane protein KASH5 are depicted. KASH5 binds to the dynein–dynactin complex, which runs on microtubules. **b** Ultrathin section of a pachytene spermatocyte showing telomere chromatin (Ch) of the homologous chromosomes associated with the synaptonemal complex (SC) at the nuclear envelope (NE). **c** Telomere attachment site of epoxy-embedded frozen testis section. Lateral elements (LE, magenta) and central element (CE, yellow) of the synaptonemal complex, attachment plates (AP, purple), and nuclear envelope (NE, green) are highlighted. Arrowheads point at filaments originating from the inner nuclear membrane, spanning the perinuclear space and protruding into the cytoplasm. Scale bars (**b**, **c**): 100 nm

Genome stability and genetic diversity in sexually reproducing organisms is achieved through the haploidization and recombination of homologous chromosomes during meiosis. To recombine, the homologs must find each other and align along their entire length using the tripartite synaptonemal complex as a proteinaceous scaffold^1,2^. At the onset of prophase I, the meiotic telomeres attach to the nuclear envelope and move along its plane until they encounter and pair with their respective partner. Telomeres actively move during leptonema, zygonema, and pachynema^3–5^. They promote stable pairing between homologs for recombination events and are therefore essential for meiotic progression^6,7^. One particularly conserved aspect of chromosome movement are the protein complexes that transduce the necessary force onto the telomeres^8^. Linker of nucleo- and cytoskeleton (LINC) complexes span the nuclear envelope and transduce cytoskeleton-derived forces into nuclei^9,10^. LINC complexes are expressed ubiquitinously and engage in a variety of functions such as nuclear migration, nuclear integrity, and chromosome movement^9–12^. The LINC complex comprises the type II inner nuclear membrane Sad1 and UNC-84 (SUN) homology proteins and outer nuclear membrane Klarsicht, Anc1 and Syne 1 homology (KASH) domain proteins. In mammals, at least five SUN and six KASH proteins are encoded. SUN and KASH proteins form a 3:3 hetero-hexamer via their eponymous C-terminal domains in the periplasm. The carboxy-terminal region of the SUN protein, which resides in the perinuclear space, further composes of a coiled-coil domain that enables its trimerization^13^. Moreover, this coiled-coil domain is characteristic for structural proteins under the stress of forces, as these domains provide high elasticity to the molecule^14–16^. The meiotic LINC complex is comprised of SUN1/2 and KASH5^17–21^. The short N-terminal sequence of SUN1/2 interacts with lamin and telomere-binding proteins in the nucleoplasm while the large N-terminus of KASH5 carries a dynein-binding domain. In most eukaryotes, including mice, LINC complexes are connected to dynein–dynactin moving on microtubules^8,19,22^. A schematic overview of the hexameric nature of the SUN1/2 complex and its nucleoplasmic connection to the telomeric DNA bound by telomere-binding proteins (TERB1, TERB2, TRF1, and Majin), as well as their connection to the dynein–dynactin motor system in the cytoplasm is shown in Fig. 1a. Pharmacological inhibition of microtubule polymerization and of dynein validated dynein motors on microtubules as the main source for meiotic telomere movement as it led to significant reduction of telomere movement. Accordingly, the knockout of one of the LINC complex proteins as conveyers of cytoskeletal forces resulted in the highly significant reduction of movement in case of SUN1 and complete absence of movement in KASH5 knockout mice^4^. The slightly less severe effect of the SUN1 knockout can be assigned to the redundant function of SUN2, forming a complex with KASH5. LINC complexes are undoubtedly essential to meiotic telomere movement. In somatic cells, they are distributed over the entire nuclear envelope. In meiosis, however, they are restricted to the regions of the nuclear envelope where the meiotic telomeres are attached. Standard widefield immunofluorescences of SUN1 and KASH5 at these sites revealed focal accumulations of the proteins to the telomere attachment sites. Structured-illumination microscopy that provides approximately twice the resolution of diffraction-limited microscopy reveals ring-like assemblies of SUN1 and KASH5^4^. In two-dimensional (2D) transmission electron micrographs, the densely distributed LINC complexes appear superimposed and cannot be resolved individually^23,24^. A better understanding of LINC complex organization at these sites requires three-dimensional (3D) nanometer resolution of their macromolecular architecture within the respective morphological context. Therefore, in this work, we prepared testis tissue samples for electron microscopic tomography through which we could obtain the necessary structural detail within the cellular environment of murine spermatocytes. Through manual segmentation of the tomograms we generated 3D models of telomere attachment sites and the associated LINC complexes. Based on these models, we were able to quantify the amount, length and width of meiotic LINC complexes. This approach granted us to analyze the distribution of the filaments at attachment sites with and without microtubules, which ultimately resulted in a first estimation of the effective forces at telomere attachment sites.

## Results

### Resolving LINC complexes at telomere attachment sites

**Fig. 2:**
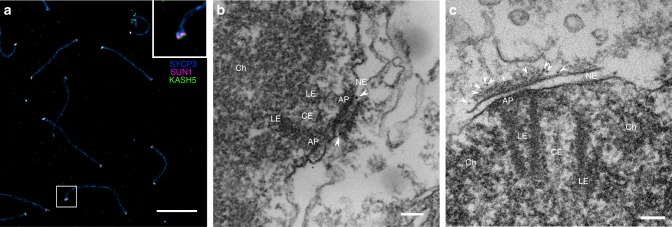
Detection of LINC complex components at meiotic telomere attachment sites. **a** SUN1, KASH5, and SYCP3 triple localization on murine testis cell spreadings imaged with structured-illumination microscopy. SUN1 and KASH5 localize to the two ends of the SYCP3 signal. Inset: magnification of one terminal SYCP3 signal revealing that SUN1 and KASH5 signals are densely packed, with the SUN1 signal likely residing in between KASH5 and SYCP3. **b** Immunogold localization of SUN1 on epoxy-embedded frozen sections. Arrowheads point to gold particles localizing to the perinuclear domain of the filaments that emanate from the inner nuclear envelope at attachment sites. **c** Immunogold localization of the KASH5 N-terminus. Colloidal gold bound to the end of the cytoplasmic filaments is indicated by arrowheads. LE: lateral element, CE: central element, AP: attachment plate, Ch: Chromatin, NE: nuclear envelope. Scale bars: (**a**): 2 µm, (**b**, **c**): 100 nm

The objective of this work is the 3D resolution and quantification of LINC complexes at meiotic attachment sites during telomere movement. Prophase stages that are characterized by telomere movement are leptonema, zygonema, and pachynema^3–5^. Pachynema is especially suited for an electron microscope (EM) tomography-based study of the telomere attachment sites as they are easily identified through the characteristic tight association of synaptonemal complexes with the nuclear envelope at this prophase stage. Therefore, we focused on analyzing LINC complexes of pachynema nuclei (Fig. 1b). The synaptonemal complexes (composed of two lateral elements and a central region) are attached to the nuclear envelope via the attachment plates, i.e., the electron dense structures at the end of the lateral elements^24^. The composition of attachment plates is poorly understood. It appears that they lack protein components of the synaptonemal complex^24,25^. On the other hand, electron microscopical in situ hybridization has revealed the presence of telomere repeats^24^. From the attachment plates, an assortment of filaments appear to originate. These filaments are anchored in the nuclear envelope, span the perinuclear space, and protrude into the cytoplasm^23,24^. Already in 2D electron micrographs, it becomes apparent that filaments concentrate at the telomere attachment sites (Fig. 1c). Yet, the number, length and distribution of the filaments remains elusive. Based on the location, the inherent function of connecting the nucleus with the cytoplasm and on previous immunogold localizations of SUN2 at these sites^17^, these filaments are likely LINC complexes. To confirm the molecular identity of the filaments immunolocalization experiments were conducted using SUN1 and KASH5 antibodies. In a triple immunofluorescence staining for SUN1, KASH5, and SYCP3 (a major component of the lateral elements) on meiotic chromosome spreadings, the LINC complex proteins localized at the end of the synaptonemal complex (Fig. 2a). This signal distribution is in agreement with previous immunolocalization experiments, in which antibodies, directed against different epitopes of the proteins were used^5,20^. Image acquisition of the triple staining with structured-illumination microscopy indicates SUN1 (as an inner nuclear membrane protein) localizing in between SYCP3 and KASH5, which is in agreement with previous studies^5,20^ (inset Fig. 2a). However, confirmation that the filamentous structures at telomere attachment sites in the micrographs are indeed LINC complexes demands the molecular mapping of SUN1 and KASH5 to these filaments at higher resolution in structurally well preserved testis tissue. To this end, we performed pre-embedding immunogold electron microscopy of SUN1 and KASH5 on frozen testis sections. SUN1 labeling resulted in gold particles localizing to the part of the filaments in the perinuclear space between the inner and outer nuclear membranes (Fig. 2b). This localization is in agreement with previously published immuno-EM localizations of SUN2 and immuno-EM localizations of SUN1 shown in the Ph.D thesis of Johannes Schmitt of the University of Wuerzburg^26^ at murine telomere attachment sites. SUN2 replaces SUN1 in the hexameric LINC complex and functions redundantly to SUN1. Immunogold labeling against the N-terminus of KASH5 resulted in colloidal gold being bound exclusively to the cytoplasmic end of the filaments (Fig. 2c and Supplementary Fig. 1a). Gold particles did not localize to the end of the filaments when the sections were only incubated with the secondary antibody conjugated to 6 nm gold but not with the primary antibody against the N-terminus of KASH5 (negative control in Supplementary Fig. 1b). To our knowledge, this is the first immuno-EM localizazion of KASH5. We confirmed the localization of the N-terminus of KASH5 to the end of the filaments in over ten telomere attachment sites. Hence, our result provides clear evidence that the filamentous structures concentrating at telomere attachment sites, which connect the nuclear interior with the cytoplasm are indeed LINC complexes.

### EM tomography of LINC complexes at telomere attachment sites

EM tomography allows for the three-dimensional resolution of macromolecular protein complexes in their morphological context. Recent technical advances such as high-pressure freezing and freeze substitution allow for close-to-native structural preservation of electron microscopic samples through rapid fixation by vitrification and gradual exchange of cellular water at low temperatures. Mouse testes however display very little intertubular connective tissue^27^. Therefore, intracellular cohesion is weak and tissue integrity easily lost due to shearing forces during high-pressure freezing. To stabilize the tissue prior to vitrification, we chemically fixed the individual seminiferous tubules^28^. In combination with subsequent high-pressure freezing and freeze substitution, this refined protocol resulted in excellent ultrastructural preservation, a prerequisite for quantitative EM tomography (Supplementary Fig. 2)^29^. As mentioned earlier, pachynema spermatocytes are ideally suited for electron microscopic studies of meiotic LINC complex assemblies. At this stage, telomeres are motile and attachments as sites of LINC complex accumulation easily identified at the electron microscope. Furthermore, the first spermatogenesis wave progresses rather synchronously so that in testes of 14-day-old mice early to mid pachynema spermatocytes are found. Later meiocyte stages are absent at this age. Synchronous development of same stage germ cells allows for direct comparison between individual pachynema spermatocytes.

**Fig. 3:**
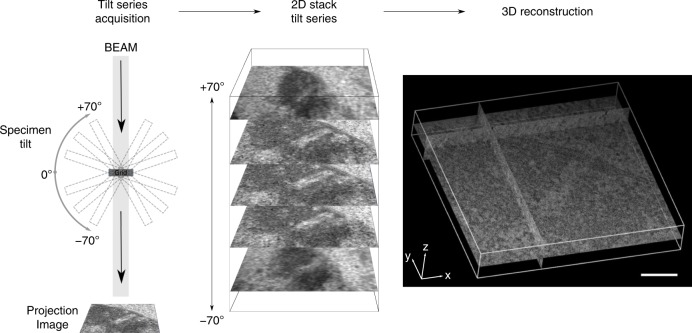
Tilt series acquisition and tomogram reconstruction of meiotic telomere attachment sites. One-hundred forty-one images of the telomere attachments are acquired by tilting the sample in one degree steps from −70° to +70°. The 2D stack of the projected images is back-projected to reconstruct the originial volume to a tomogram comprised of virtual sections. Scale bar: 200 nm

**Fig. 4:**
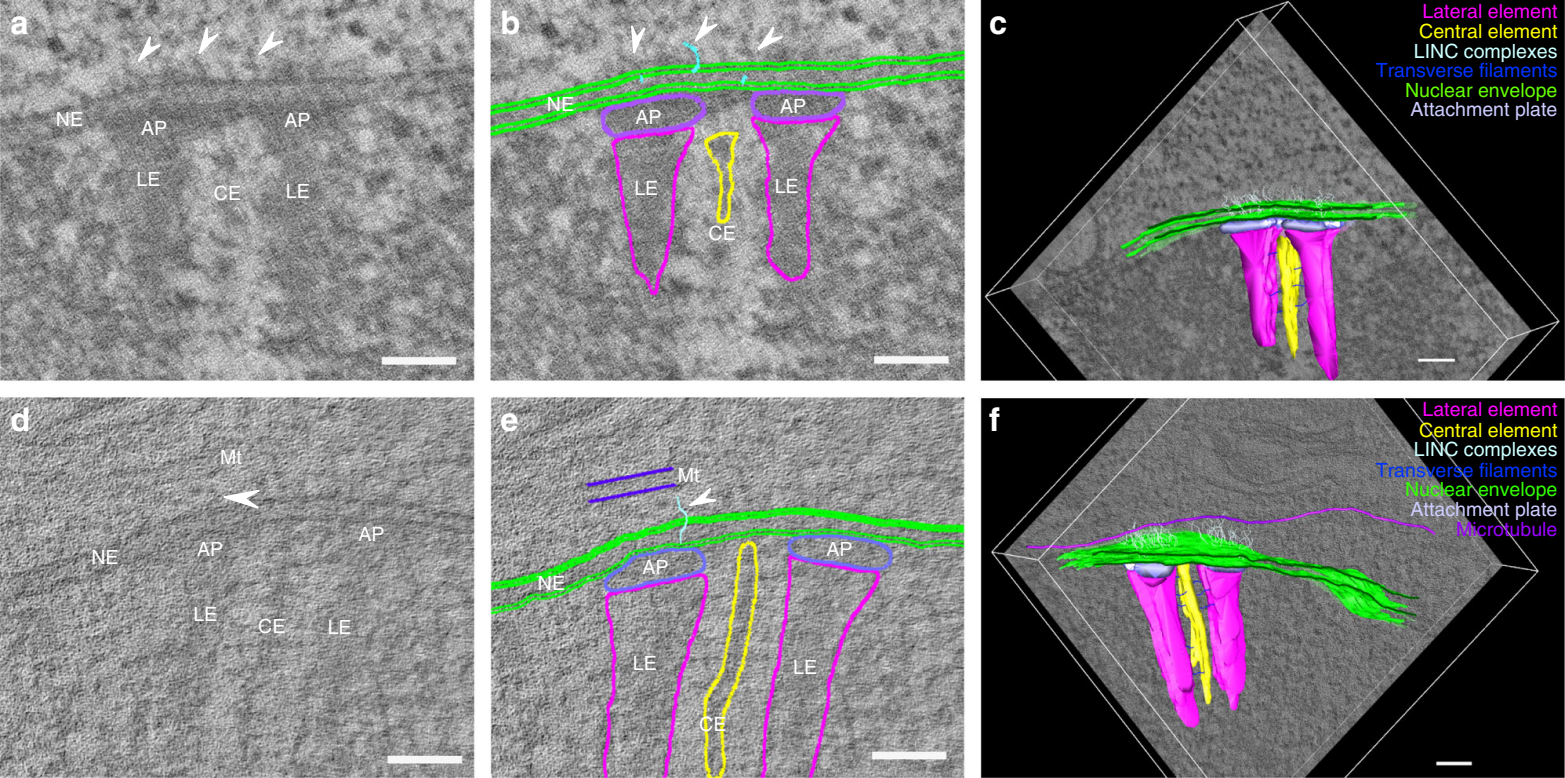
Segmentation for 3D model generation of telomere attachment sites. **a**, **d** Single virtual section of a reconstructed tomogram of a telomere attachment site without a microtubule (**a**) and with a microtubule running parallel to the frontal view of the synaptonemal complex (**d**). **b**, **e** Respective manual segmentation of the virtual sections of **a** and **d**. **c**, **f** Resulting 3D models of telomere attachment sites from the combination of all individual segmentations. LE: lateral element, CE: central element, AP: attachment plate, Ch: Chromatin, NE: nuclear envelope, Mt: microtubule. Arrowheads indicate LINC complexes associated with the attachment sites. Scale bars: 100 nm

In a frontal view, a telomere attachment site is about 400 nm wide and up to 200 nm deep (see below). To resolve the entire attachment with all associated LINC complexes in a single preparation we prepared 250 nm semi-thin section for EM tomography. These relatively thick sections are imaged from different angles at a transmission electron equipped with a mechanical stage. The stage is tilting the sample at eucentricity in 1° increments in a range from −70° to +70°. At each tilt angle an image is acquired, which results in 141 images total, each containing the whole telomere attachment site, including cytoskeletal components imaged from a slightly different angle. After the completion of this first tilt series, the sample is rotated by 90 degrees and reintroduced to the microscope. A second tilt series is acquired that leads to a combined *z*-stack of 282 two-dimensional images of the double tilt. Full 360° resolution of samples is impossible to this day because maximum tilting of mechanical stages is limited to 70°. The 2D tilt series stack is reconstructed to a 3D volume of original dimensions using the weighted back-projection algorithm of etomo. The recombined tomogram is composed of an assembly of individual virtual sections making up the initial volume (Fig. 3). An initial visual inspection of the tomograms reveals the extensive nature of the the two ribbon-like lateral elements surrounding the central elements on both sides. Owing to the excellent structural preservation the inner and outer nuclear membranes maintain a regular spacing throughout the section. The attachment plates appear as slightly darker disks closely associated with the inner nuclear membrane envelope. Owing to the three-dimensional properties of the LINC complexes whole individual filaments are hardly detected in a single section. However, by moving through the stack of virtual sections their trajectory in *z* is easily traceable. Through sole visual inspection of the tomograms, it becomes clearly apparent that a dense assortment of LINC complexes exclusively emanate from the distinct parts of the nuclear envelope, which are associated with the attachment plates. The nuclear envelope section in between the attachment plates virtually lacks filaments. Hence, two aggregations of LINC complexes per attachment site can be distinguished (Fig. 4a, d). A quantitative analysis of these protein assortments demands for a 3D model of the attachment site components.

### Visual inspection and supervised LINC complex segmentation

For this study, 11 tomograms of telomere attachment sites were acquired. Visual inspection of the volumes revealed that five of these tomograms contain a single microtubule close to the respective attachment site. The microtubules were either oriented longitudinally or transversally to the frontal view of the attachment. The tomograms were manually segmented for the respective features of interest. In each of the virtual tomogram sections the lateral and central element, the attachment plates, the inner and outer nuclear envelope, as well as the LINC complexes were traced (Fig. 4b, e). Only some of the transverse filaments that connect the two lateral elements with the central element were annotated to document that they can also be resolved under these experimental conditions. (As a characterization of the transverse filaments is not the subject of this work, we did not engage in an extensive segmentation of the whole set of transverse filaments). LINC complexes were segmented according to pre-defined criteria to minimize subjective bias during segmentation. LINC complex origins were assigned to their location in the inner nuclear membrane. From the inner nuclear membrane, filaments were traced through the perinuclear space into the cytoplasm based on continuity. As mentioned earlier, the three-dimensional nature of these filaments makes a depiction of an entire LINC complex in a single virtual section rare. Supervised segmentation always demands for simultaneous visual tracking of the filaments through the stack of virtual sections during annotation. This ensures the accurate detection and subsequent 3D representation of LINC complexes at a telomere attachment site in three-dimensional models assembled from the segmentations of the individual virtual sections (Fig. 4c, f and Supplementary Movies 1 and 2; high-resolution movies available at ref. ^30^). These 3D models allow for a quantification of the LINC complexes at these sites. For the analysis, attachment sites were assigned to two separate groups: with and without microtubule.

### Quantification of LINC complexes at attachment sites

**Fig. 5:**
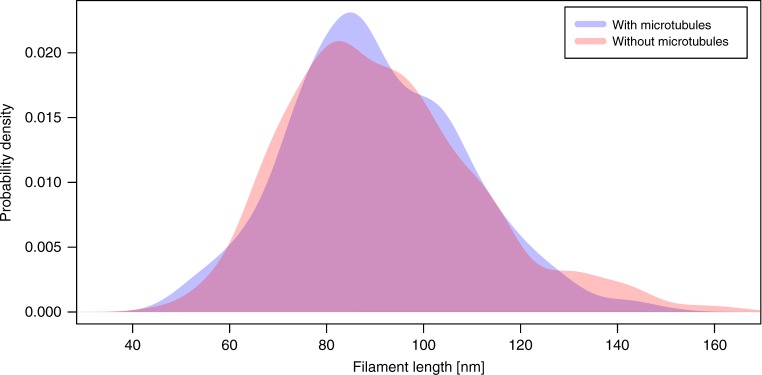
Density curves describing the distribution of the filament lengths

The amount and length of the LINC complexes can directly be extracted from the 3D model with the IMOD console program imodinfo. Table 1 gives an overview of the number of filaments per attachment site. It further indicates whether the respective attachment was associated with a microtubule. On average, 76 LINC complexes were originating from the inner nuclear membrane at the analyzed attachments. There is no significant difference between the two data sets (two-sample Kolmogorov–Smirnov test, *p* = 0.969). The filaments are not branching and apparently not interconnected with each other. The length of the filaments corresponding to LINC complexes is roughly 90 nm on average. Filaments at attachment sites with a microtubule have a mean length of 92 ± 20 nm. In the absence of a microtubule, LINC complexes are 91 ± 18 nm long. A two-sample *t*-test with a *p*-value of 0.078 confirms that the filament length as the number does not significantly differ between the two subsets of attachments. We analyzed the distribution of the filament length data in more detail in order to depict the possible existence of subpopulations of different lengths at the attachment sites. To analyze the length data for such subgroups, we additionally plotted the lengths as density distributions (Fig. 5 and Supplementary Data 2). Both density curves (with/without microtubules) reveal that the length data is distributed around the one single mean length of roughly 90 nm that we determined. There are no other local maxima. Hence, filaments at telomere attachment sites cannot be grouped in longer and shorter filaments. As mentioned above, LINC complexes are comprised of SUN1/2 and KASH5 in mouse. In 2012, Sosa et al.^13^ proposed a length of 45 nm for the coiled-coil of SUN1/2. KASH5 consists of 200 amino acids. Consequently, SUN1/2 and KASH5 together are likely not exceeding 100 nm in length. The density distribution of the LINC complex lengths we measured show outliers of both shorter and longer filaments (Fig. 5). The main criterion of segmentation in this study has been continuity of the filaments. Occasionally, in the crowded environment of the cytoplasm, filamentous proteins appear to be continuous with LINC complexes. We thus cannot avoid tracing longer outliers. Overall, the segmentation is a statistical method and the 832 LINC complexes traced in this study reliably reflect the properties of the LINC complexes. Apart from average number and length of filaments we also determined their width. The filament width of a single LINC complex was measured at four designated positions, which were subsequently averaged to give a fair representation of the overall LINC complex width. The resulting values of these diameters were used to calculate an overall average filament width of 2 nm for LINC complexes at telomere attachment sites (filaments of attachments with microtubule: 1.9 ± 0.3 nm; filaments of attachments without microtubule: 1.9 ± 0.2 nm).

**Table 1:** Overview of the number of filaments of each analyzed attachment and closeness of a microtubule

Attachment	Microtubule	Number of filaments
1	No	69
2	No	104
3	No	77
4	No	64
5	No	63
6	No	80
7	Yes	80
8	Yes	67
9	Yes	79
10	Yes	88
11	Yes	61

### LINC complex distribution at telomere attachment sites

**Fig. 6:**
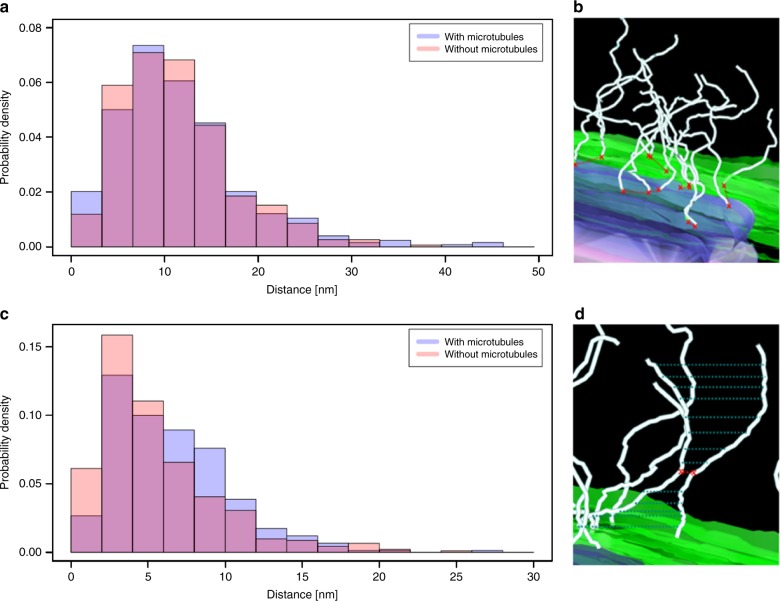
Histograms of LINC complex distributions at meiotic telomere attachments to the nuclear envelope. Minimum distances between filament origins (**a**, **b**) and minimum interfilamentous distances of two neighboring filaments (**c**, **d**) are shown. The respective distances are indicated by red crosses and red dashed lines in the magnified view of the attachments (**b**, **d**). Blue dashed lines in **d** illustrate that distances between all point coordinated of the neighboring filaments were measured. Inner nuclear membrane (green), attachment plate (purple), LINC complexes (light cyan)

**Fig. 7:**
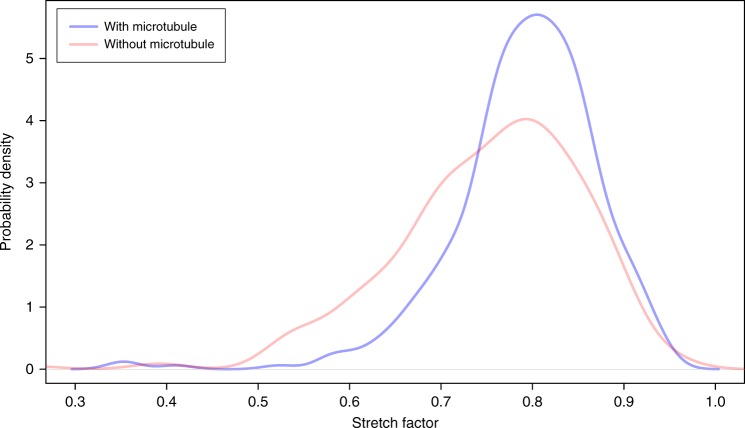
Stretch factor of LINC complexes. Density plot of the stretch factor (linear distance/length) of LINC complexes at attachment sites close to a microtubule and those without a microtubule

**Fig. 8:**
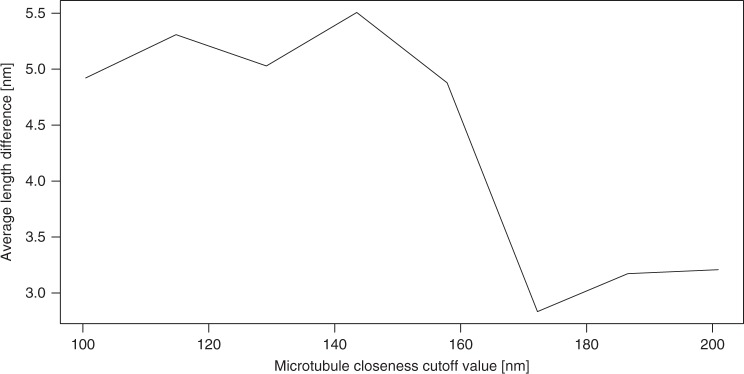
Difference in average linear distance between filaments close to a microtubule in close proximity to and far from the microtuble for varying cutoff values

The load-bearing properties of LINC complexes and dense distribution of the fibrillar proteins at the attachment sites raise the question whether they are organized according to a specific pattern that facilitates their function. We first analyzed the distribution of the apparent origin of LINC complexes at the inner nuclear envelope by calculating the minimum distance between neighboring filament origins at this site. The point coordinates of the individual filaments were extracted using the command line application model2point of the IMOD software suite. To determine the origins of the filaments from this set of points we calculated the Euclidean distance between the point coordinates of the individual filaments and the point data of the lateral element model points. Euclidean distance hereby refers to the root of square differences between the coordinates of two points in 3D. The coordinate with the minimum Euclidean distance to the lateral element points corresponds to the filament origin. We then calculated the minimum Euclidean distance between the individual filament origins and plotted them as a histogram (Fig. 6a, b). The curve resembles an exponential distribution, which indicates that there is no common distance between neighboring filaments. However, the exponential function is shifted to the right by 5 nm from the origin of the *x-*axis, which suggests a global minimum distance between neighboring filament origins of 5 nm. There was no significant difference in data behavior between filaments at attachments with a microtubule to those without (Fig. 6a; two-sample Kolmogorov–Smirnov test, *p* = 0.079; Supplementary Data 3). Additionally, using principle component analysis, we determined the size of the area where the LINC complexes concentrate at the nuclear envelope. We fitted a plane to the LINC complexes origins associated with one attachment plate. The side of the plane, which is parallel to the nuclear envelope, is roughly 150 nm long. In a next step, we determined the minimum distances between the entire lengths of two neighboring LINC complexes (Fig. 6c, d). We therefore calculated the minimum Euclidean distances between all point coordinates of the two filaments being compared. Again, this analysis was performed separately for the attachments without and with microtubule motor sytem association. The respective histograms reveal that the minimum distances between the entire lengths of neighboring filaments are described by two curves resembling an exponential distribution, which is shifted right from the *x*-axis origin by 2 nm, reflecting the global minimum for both data sets (Fig. 6c and Supplementary Data 3). Notably, there is a significant difference of the average minimum filament distance between the two distributions (two-sample Kolmogorov–Smirnov test for equality, *p* = 2.66 × 10^−5^). The average minimum filament distance is larger for LINC complexes, which are associated with microtubules. A possible reason for the larger interfilamentous distance of microtubule-associated filaments could be a different degree of curvature of these LINC complexes. To test this hypothesis, we calculated the stretch factor (linear distance/length) for LINC complexes at attachment sites with a microtubule and compared it to LINC complexes at attachment sites without a microtubule. We generated a density plot of the respective stretch factors, which revealed that the stretch factor of LINC complexes close to a microtubule is indeed larger (Fig. 7 and Supplementary Data 4). This larger stretch factor reflects a less rippled state of LINC complexes at attachment sites with a microtubule, in comparison to LINC complexes at attachment sites without a microtubule. We additionally investigated this point by calculating the linear distance between the ends of a LINC complex. This linear distance was 3.5% greater on average for filaments close to a microtubule (LINC complexes at attachment sites with a microtubule: 72.6 nm; attachments without a microtubule: 70.1 nm). Since only a fraction of these filaments is in close proximity to the microtubule at the time of fixation, the detectable effect on filament curvature might be decreased in these measurements. For a more fine-grained analysis, we therefore divided the LINC complexes at attachment sites with a microtubule in two groups: filaments far away from and close to the microtubule. Figure 8 shows the difference in the average linear distance between the filament end points according to their distance to the microtubule at different cutoff values defining proximity to the microtubule (Supplementary Data 5). LINC complexes that are <100 nm away from the microtubule are about 4 nm longer (linear distance) opposed to the filaments above this cutoff value. According to the Wilcoxon rank-sum test this difference is statistically significant. The length difference remains similar for cutoff values up to 158 nm and steadily decreases afterwards. In other terms, LINC complexes close to a microtubule (up until 160 nm) appear to be less rippled and therefore stretch further from the nuclear envelope (NE).

## Discussion

Meiotic chromosome movement throughout prophase I is essential for establishing genetic diversity in sexually reproducing organisms. The mutual recognition and pairing of nuclear envelope attached homologs is a prerequisite for successful recombination, as well as their subsequent segregation^31^. Meiotic chromosome movements facilitate the recognition and pairing of the homologs. The movements hereby depend on the transduction of predominantly microtubule motor system-derived forces onto meiotic telomeres through LINC complexes^4,18–20^. Consequently, a resolution of the molecular architecture of the LINC complexes at meiotic telomere attachment sites would provide a better understanding of the mechanisms propelling synapsis and recombination. To this end, we have resolved the entire set of LINC complexes at these sites with EM tomography in murine pachytene spermatocytes. We subsequently generated 3D models of the telomere attachment sites through manual segmentation of the tomograms. Based on these models we provide quantitative and topological data on the meiotic LINC complexes.

On average, 76 filaments with a mean length of roughly 90 nm and a width of 2 nm localize exclusively to the two distinct attachment plates at the nuclear envelope of a telomere attachment site. Our EM tomography results on LINC complex distribution at telomere attachment sites in pachytene do not support structured-illumination microscopic observations describing a ring-like distribution of SUN1 and KASH5 acquired at the same prophase stage with a central area lacking LINC complexes. Our results rather favor the notion that these ring-like assemblies might be an artifact due to antigen masking or reduced accessibility to the center of the LINC complex clusters^20^. A change in LINC complex distribution might, however, occur depending on the prophase stage. While early prophase stages (leptotene and zygotene) are characterized by fast telomere movements aimed at the pairing of the homologs, the speed of telomere movements decreases in later prophase stages (with pachytene obtaining the slowest pace), when homolog pairing is complete and recombination occurs^4^. It is possible that these different processes and the related speeds entail the need for different LINC complex distributions linked to the respective prophase stage. Along the same lines, we hypothesized that the stage-specific difference in telomere movement speed could further impede the need of the attachment sites to be connected to the microtubule motor system all together. Previous confocal microscopical immunolocalization of SUN1 had suggested that nearly all telomere attachment sites colocalize with tubulin, which makes up the few microtubules surrounding the nuclear envelope^4^. However, the higher resolution provided by electron microscopy in this study revealed that only a subpopulation of telomere attachment sites are close to a microtubule at a certain time point. Furthermore, our EM tomography data reveal the presence of a single-microtubule per telomere attachment site that runs predominantly parallel to the synaptonemal complex frontal axis. The presence of a microtubule at certain telomere attachment sites and the corresponding absence at others suggests that the former sites were potentially moving at the time of fixation. Accordingly, telomere attachment sites without a microtubule were likely immobile. These findings are in agreement with live-cell imaging and confocal immunolocalization of murine prophase nuclei, which show that telomeres are not permanently moving but also pause/stop^3,4,31^. Telomeres close to a microtubule contain the same average number of LINC complexes as their stationary counterparts. Consequently, filaments appear to be constitutive components of the telomere attachment site. Unfortunately, our approach does not allow the visualization of dynein at the meiotic attachment sites. Therefore, we cannot refer to a direct association between the LINC compexes and the motor system connected to microtubules in our tomograms. However, the microtubule in the individual tomograms runs exactly through the accumulation of LINC complexes at the respective attachment site with a subset of complexes appearing to directly touch the microtubule. In 2015, Lee et al.^4^ provided experimental evidence on the association of LINC complexes with the microtubules via dynein at these sites. Moreover, we report differences in the stretch factor of LINC complexes between the attachments sites with microtubules compared to those without. We suggest that this difference stems from the association of LINC complexes with microtubules through dynein as further discussed below. A mouse diploid cell has 40 chromosomes and therefore 80 telomeres. In pachynema, homologous chromosomes are fully synapsed via the synaptonemal complex forming a bivalent. In the case of autosomes, each bivalent spans the meiotic nucleus and the two pairs of telomeres are anchored at different sites of the nuclear envelope. (The XY bivalent is an exception, as synapsis is partial, resulting in three attachment sites). Thus, with an average of 76 filaments per telomere pair, we calculate a total amount of ~3000 filaments for a murine meiotic nucleus. Remarkably, these results provide a first calculation of the total amount of LINC complexes that would be involved in the transduction of forces from the cytoplasm into the nucleus in a certain cell type. Moreover, since LINC complexes appear to be formed of trimers of SUN and KASH proteins^13^, <10,000 SUN1/2 and 10,000 KASH5 molecules per nucleus would be involved in the mentioned functions.

In addition to the quantitative analysis of the LINC complexes, the 3D models also allowed us to analyze the topological organization of the filaments at telomere attachment sites. LINC complex origins at the inner nuclear membrane have a global minimum distance of 5 nm, while the distances between the entire lengths of two neighboring filaments showed a global minimum of 2 nm (Fig. 6a, b). Interestingly, the average distance between neighboring LINC complexes of attachment sites associated with a microtubule was larger (Fig. 6b). This could be explained with a less rippled conformation of these proteins. Measurements of a significantly larger linear distance and moreover a larger stretch factor for LINC complexes in close proximity to the microtubule strongly suggest that LINC complexes are in a less rippled conformation when in contact with the cytoskeleton. We propose that this condition could be related to the interaction of filaments with microtubules and associated motor proteins.

Dynein–dynactin complexes moving on microtubules are essential for telomere prophase movements^4,18–20^. The amount of force required to move a pair of meiotic homologs on the plane of the NE remains elusive. The 3D model presented here allows some speculations on this aspect. We have shown that a microtubule interacts with LINC complexes over a length of about 150 nm two times per attachment site. Recently, Lander and colleagues^32^ determined the dimensions of the microtubule-bound dynein–dynactin complex that is ca. 43 nm long. Assuming that the dynein–dynactin complexes are packed at maximum density on a microtubule, up to eight complexes could be accommodated per telomere attachment site. The stoichiometry between the maximum of eight dynein–dynactin complexes and the roughly 80 LINC complexes per attachment site remains elusive. Since a single dynein–dynactin complex exerts a force of ~5 pN^33^, about 40 pN could be generated per telomere attachment site in pachynema. A more recent study resolved the 3D structure of a murine microtubule-bound dynein–dynactin complex in brain tissue with a cryo-electron tomographic approach. The authors showed that a single dynactin can bind two dynein motors, increasing the force generated by a single dynein–dynactin complex by a factor of two^34^. In such a case, about 80 pN could be generated at a single-telomere attachment site.

There is strong evidence that dynein–dynactin-binding adaptor proteins such as BICD2 and HOOK3 are responsible for whether one or two dynein motors are part of a motor protein complex. Association of HOOK3 causes two dynein motors to be associated with dynactin. BICD2 can mediate complex formation with either one or two dyneins^34,35^. In mammals, BICD2 facilitates vesicle transport and positions the nucleus in a microtubule dependent fashion. For the latter, BICD2 is recruited to the nuclear envelope by the nucleoporin RanBP2. BICD2 is the only dynein adaptor that associates with membranes, including the nuclear envelope. Consequently, it is of special interest in a study of nuclear envelope residing LINC complexes and their connection to the microtubule motor system^36^. In the Ph.D thesis of Anna Salter of the University of Manchester from 2016 the role of KASH5 as a potential dynein adaptor and its relation with the dynein adaptor BICD2 has been studied in depth. In co-immunoprecipitation experiments, BICD2 does not precipitate with KASH5. Both proteins bind to dynein in the same region (but not the same domain) and compete in forming a stable tripartite complex with dynein. On the basis of this competition between KASH5 and the dynein adaptor BICD2, KASH5 has been suggested to be a dynein adaptor protein^37^. Whether KASH5 recruits one or two dyneins to the dynein–dynactin complex is unknown. Therefore, both 40 and 80 pN are possible upper limits for forces generated at telomere attachment sites. This first approximation is intended to provide a basis for future experiments measuring mechanotransduction of forces via LINC complexes directly.

## Methods

### Animals

Wild-type C57BL/6J mice were housed and bred in accordance to the German Animal Welfare Act. Prior to testis resection, animals were euthanized with CO_2_ followed by cervical dislocation. Animal care and experiments were conducted in accordance with the guidelines provided by the German Animal Welfare Act (German Ministry of Agriculture, Health, and Economic Cooperation). Animal housing and breeding was approved by the regulatory agency of the city of Würzburg (Reference 821-8760.00-10/90 approved 05.06.1992; according to §11/1 No. 1 of the German Animal Welfare Act).

### Immunogold electron microscopy on frozen testis sections

Wild-type testes of 8–12-week-old male C57BL/6 mice were fixed for 1h  with 1% paraformaldehyde in phosphate-buffered saline (PBS), then covered in Tissue-Tek embedding medium and transferred into methyl butane cooled by liquid nitrogen. The frozen tissue was cut with a cryo-microtome (2800 Frigocut E, Reichert-Jung) into 9 μm sections and dried down to poly-lysine slides. Sections were rinsed in PBS, rehydrated for 15 min in 25 mM ammonium chloride to block free aldehyde groups after fixation, and then washed with PBS. After permeabilization with 0.05% triton X-100 for 10 min, unspecific epitopes were blocked with phosphate-buffered saline and Tween 20 (PBT) for 1 h. Primary and secondary antibodies were diluted in PBT (SUN1 1:50, KASH5 1:200, 6 nm gold anti-rabbit/guinea pig 1:10 from Dianova, Hamburg, Germany), primary antibodies were additionally centrifuged at 16,000 × *g* for 10 min at 4 °C. After incubating the tissue with the primary antibody for 1 h, epitopes were blocked for another hour with PBT. Sections were washed with PBS before being incubated with the secondary antibody for 1 h. For the negative control of the KASH5 N-terminus samples were processed accordingly with the difference that the primary antibody incubation was skipped and sections were solely incubated with the secondary antibody. Post-fixation was carried out for 35 min with 2.5% glutaraldehyde in 50 mM cacodylate buffer and subsequently with 2% osmium tetroxide for 1 h. Prior to dehydration, sections were washed with ddH2O. Sections were dehydrated in an ascending ethanol series (50%, 70%, 90%, 100%, 100%) on ice for 5 min each. The tissue was then incubated two times for 5 min in 100% propylene oxide before it was infiltrated with a 1:1 mixture of propylene oxide and epoxy resin over night at room temperature. The resin was exchanged with 100% epoxy resin after 16 h and infiltrated for another 4 h before the sections were embedded in fresh epoxy resin and curated for 48 h at 60 °C. Glass slides were split off after immersion in liquid nitrogen using pressured air. Ultrathin sections were then cut using a Histo Jumbo Diamond Knife (Diatome, Biel, Switzerland) to 50–65 nm sections. The latter were transferred onto formvar coated slot grids and contrasted with 2.5% uranyl acetate in ethanol for 15 min and 50% Reynold’s lead citrate in ethanol for 10 min before introducing them to the transmission electron microscope.

### Antibodies

To detect the N-terminus of KASH5, two peptides (aa 71–83, aa 89–104) against murine KASH5 were synthesized and used to co-immunize a rabbit. The resulting polyclonal antibody was purified by sulfolink affinity chromatography (BIOTEM, Apprieu, France). Other primary antibodies used in this study: guinea pig anti-SUN1^38^ (aa 427–722) and mouse monoclonal anti-SYCP3 (Abcam; ab97672).

### Immunofluorescence microscopy

Spermatocyte cell spreadings of male 14-day-old wild-type C57BL/6J mice were prepared according to the protocol described by de Boer et al.^39^. Briefly, testes were resected and transferred to PBS. Seminiferous tubuli were removed from the testes and incubated in hypotonic buffer (30 mM Tris/HCl, 17 mM sodium citrate, 5 mM EDTA, 50 mM sucrose, 5 mM DTT) for 1 h. Cells were then extracted by disruption of swollen tubuli in 100 mM sucrose and spread in a drop of 1% paraformaldehyde with 0.15% Triton X-100. The spreads were incubated in a wet chamber for 2 h and dried down on poly-lysine slides over night in the wet chamber without the lid. Slides were stored at −80 °C in aluminum foil and thawed upon usage. For the immunofluorescence and subsequent structured-illumination imaging, spreads were washed with PBS. Unspecific epitopes were blocked with 10% normal goat serum for 1 h. Primary antibodies of the triple labeling experiments were incubated sequentially (SYCP3 anti-mouse 1:100, SUN1 1:100, KASH5 1:200) for an hour each. Following washing of the spreadings with PBS, epitopes were blocked with 10% normal goat serum. Secondary antibody incubation was carried out sequentially for 30 min per antibody (dilution: 1:200; F(ab’)2 anti-mouse SeTau, IgG anti-guinea pig 568, F(ab’)2 anti-rabbit 488; Thermo Fisher, Langenselbold, Germany). After washing with PBS, the samples were embedded in ProLong Glass anti-fade mountant (Thermo Fisher, Langenselbold, Germany).

### Structured-illumination microscopy (SIM)

Patterned illumination was carried out on an ELYRA S.1 super-resolution structured-illumination microscope (Zeiss). *z*-stacks (five rotations, five phase shifts) of at least 1 µm at a *z*-step interval of 100 nm were acquired. Images were reconstructed using the SIM algorithm of the Zeiss Zen 2 software 10.0 black edition. Subsequent channel alignment was performed based on a transformation matrix generated from reconstructed images of 0.1 µm TetraSpek beads (*z*-step: 90 nm, *z*-range: 2 µm). Chromatic aberration corrected, reconstructed images were further processed in ImageJ 1.52e: maximum intensity projection, background substraction with rolling ball algorithm (radius 0.3 µm), histogram stretching. Image processing was carried out on individual channels, which were merged afterwards.

### Pre-fixation and high-pressure freezing

Tissue preparation for EM tomography was performed according to well-established protocols^40^. Briefly, seminiferous tubules of male 14-day-old mice were pre-fixed using Karnovsky fixative. Tubules were then transferred into freezing platelets with 200 µm chamber depth (Leica Microsystems, Wetzlar, Germany) containing 10% bovine serum albumin (BSA) in PBS. Cryofixation was performed at a freezing speed of >20,000 K s^−1^ and >2100 bar pressure (EM HPM100 from Leica, Wetzlar, Germany). Next, the samples underwent freeze substitution based on previously published protocols^41–43^ in a freeze substitution system (Leica EM AFS2, Wetzlar, Germany; see supporting information for freeze substitution protocol: Supplementary Table 1) before they were embedded in epoxy resin that was cured for at least 48 h at 60 °C. The hybrid approach of combining chemical pre-fixation with high-pressure freezing was adapted from Dhanyasi et al.^28,29^.

### Electron microscopy sample preparation

Two-hundred fifty nanometer semi-thin tissue sections were cut using a Histo Jumbo Diamond Knife (Diatome, Biel, Switzerland) and transferred onto slot or mesh grids for electron microscopy. Samples were contrasted with 2.5% uranyl acetate (UA) in ddH2O or ethanol with incubation times of 10 min in aqueous solution and 15 min in alcohol. Samples underwent further contrasting with 50% Reynolds’ lead citrate (5 min after treatment with UA in ddH2O, 10’ in case of UA in ethanol) in ddH2O. Dried samples were carbon coated to reduce charging during electron microscopy. Samples were additionally incubated with guinea pig IgG coupled to 12–18 nm gold acting as fiducial markers in tomogram reconstruction^44,45^.

### Transmission electron microscope (TEM)

Perpendicular oriented dual-axis and single-axis tilt series of synaptonemal complex attachments of pachytene spermatocytes were acquired at a magnification of x40,000 using a JEM-2100 (JEOL, Munich, Germany). The transmission electron microscope was operated at 200 kV and image acquisition was conducted with a TemCam F416 4kx4k camera (Tietz Video and Imaging Processing Systems, Gauting, Germany). Both axes of the tilt series were recorded from −70° to +70° in 1° increments with SerialEM^44^.

### Electron tomogram reconstruction and modeling

Tilt series alignment and tomogram reconstruction based on weighted back-projection was conducted by Etomo. For 3D annotation tomograms were segmented manually using 3dmod. Both programs belong to the IMOD suite^45^. Using this approach, a total of 11 tomograms were annotated, five with and six without microtubules.

### Data processing

Average numbers of cytoplasmic filaments per tomogram as well as average filament lengths were extracted directly from 3dmod using the command line program imodinfo. For raw filament length data see Supplementary Data 1. Filament widths were measured with the measure option of the 3dmod drawing tools. A second IMOD command line program, point2model, was applied to the data to generate a point representation of the 3D model. The extracted point coordinates were imported into a custom Matlab program that first calculates the closest euclidean distances between the individual filaments. Secondly, the program extracts the point coordinates of the intersection between the inner nuclear membrane and the cytoplasmic filaments, and calculates the closest euclidean distances between these points of origin. In order to determine the linear distance between the two end points of a filament, the point coordinates extracted by point2model were imported into another custom Matlab program. The program identifies the first and the last point of a filament and calculates the Euclidean distance between them. In order to approximate the filament density at the level of the nuclear envelope, we fitted a plane on the origins of the individual filaments assigned to one of the lateral elements of an attachment using principle component analysis. We used the dimensions of this plane to further derive the maximum number of dynein–dynactin complexes on the section of a microtubule at which the filaments associated with one lateral element concentrate. We mapped the area of the plane to an approximation of the respective area on the microtubule and divided the resulting value by the dimension of a dynein–dynactin complex.

### Statistics and reproducibility

Statistical analysis was performed with either two-sample Kolmogorov–Smirnov test, two-sample *t*-test or Wilcoxon rank-sum test depending on the underlying data and differences were considered as significant while *p*-value < 0.05. Data are presented as mean ± standard deviation (SD). Filaments were repeatedly verified as LINC complexes using respective antibodies against either SUN1 or KASH5 on frozen tissues of different wild-type C57BL/6J animals. The data analysis of the segmented LINC complexes led to comparable results between tomogram samples of the same subgroup (*N* = 6 for attachment sites without microtubules; *N* = 5 for attachment sites with microtubules).

### Reporting summary

Further information on research design is available in the Nature Research Reporting Summary linked to this article.

## Supplementary information

Supplementary Information

Description of Additional Supplementary Files

Supplementary Data 1

Supplementary Data 2

Supplementary Data 3

Supplementary Data 4

Supplementary Data 5

Reporting Summary

Supplementary Movie 1

Supplementary Movie 2

## Data Availability

The data sets generated during and/or analyzed during the current study are available from the corresponding author on reasonable request.

## Code availability

Custom code used for quantitative measurements in this study is available at https://github.com/MarieChristinSpindler/EvaluationScripts-ElectronTomographyOfMouseLINCComplexesAtMeioticTelomereAttachmentSites.
