# Supplementary material for: Electron tomography of mouse LINC complexes at meiotic telomere attachment sites with and without microtubules
Source: Commun Biol. 2019 Oct 14;2:376. doi: 10.1038/s42003-019-0621-1 (PMC6791847; doi:10.1038/s42003-019-0621-1)
Supplement: Supplementary file 1 — Supplementary Information [file 42003_2019_621_MOESM1_ESM.pdf]

Supporting figures

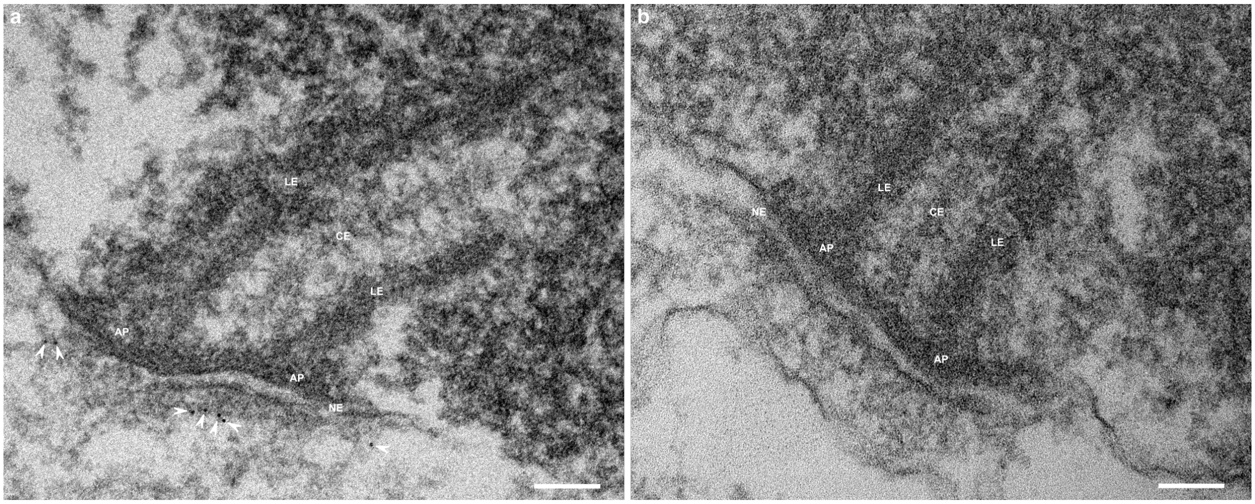

Supplementary Figure 1. Immunogold localization of KASH5 N-terminus. (A) Localization of KASH5 N-terminus on frozen sections using 6 nm colloidal gold for visualization. (B) Negative control of KASH5 N-terminus localization. Frozen sections were incubated with only the secondary antibody conjugated to 6 nm colloidal gold, without primary antibody against the KASH5 N-terminus. Note the absence of gold at the attachment site in B. Arrowheads: colloidal gold; AP: attachment plate; NE: nuclear envelope; LE: lateral element; CE: central element; Ch: chromatin. Scale bars: 100 nm.

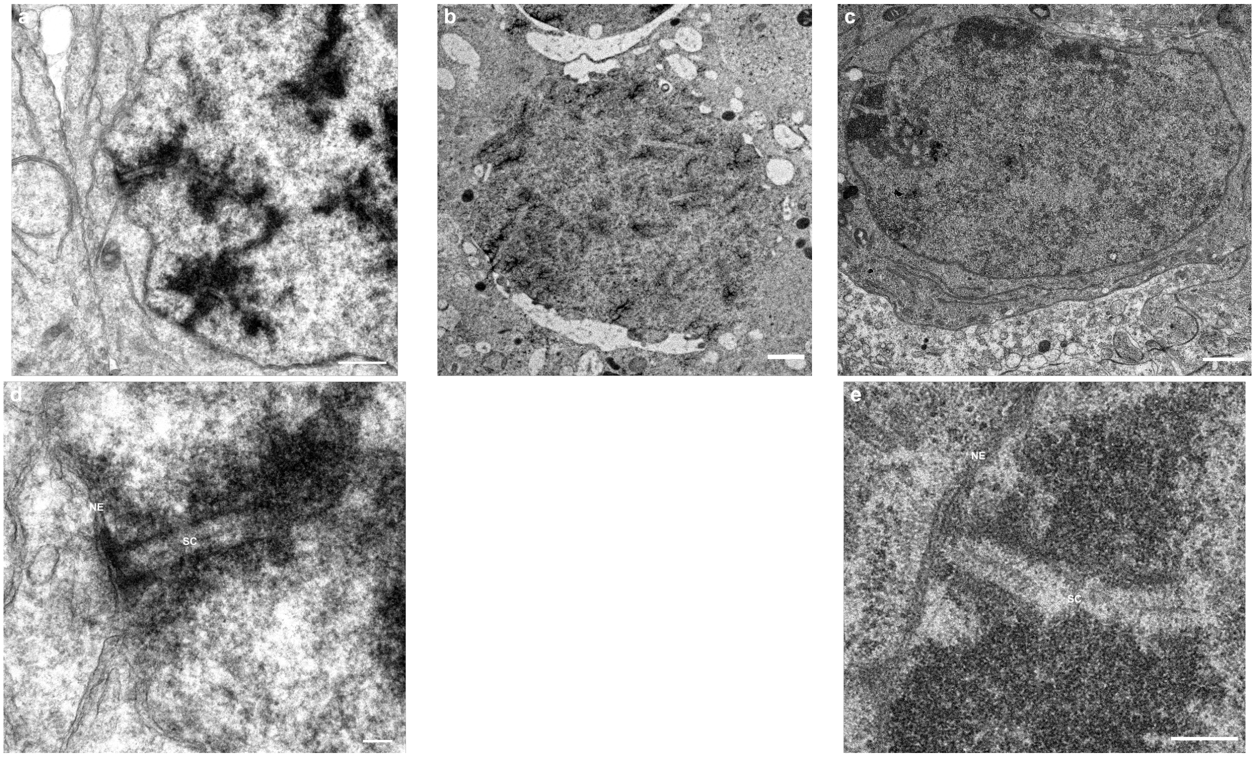

Supplementary Figure 2. Ultrastructural preservation in pachytene spermatocytes. Structural preservation after chemical fixation (A,D), high-pressure freezing followed by freeze substitution (B) and pre-fixation with subsequent high-pressure freezing and freeze substitution (C,E). A and C show an overview of the pachytene spermatocytes, D and E the respective telomere attachment site. A,D: Nuclear envelope is affected by shrinking artifacts characterized by a variation in perinuclear space. B: Nuclear content detached from nuclear envelope due to shearing forces during preparation and high-pressure freezing. C,E: Continuous even spacing of the two nuclear envelopes and overall less extracted sample indicative of a better (ultra)structural preservation. SC: synaptonemal complex; NE: nuclear envelope. Scale bars: 1 μm (A-C); 200 nm (D-E).

Supporting tables

initial temperature	final temperature	duration	solution	comment
-90 °C		96 h	0.5 % glutaraldehyde + 0.1 % tannic acid in anhydrous acetone	exchange solution once
-90 °C		4-6 h	anhydrous acetone	4 consecutive wash steps
-90 °C		28 h	2% OsO4 in anhydrous acetone	
-90 °C	-20 °C	14 h		
-20 °C		16 h		
-20 °C	4 °C	4 h		
4 °C		2-3 h	anhydrous acetone	4 consecutive wash steps
4°C	RT			remove sample pellet from carrier
RT		5 h	50 % epoxy resin in acetone	
4 °C		over night	90 % epoxy resin in acetone	
RT		2-3 h	100 % epoxy resin	
RT		2-3 h	100 % epoxy resin	
RT		2-3 h	100 % epoxy resin	
RT			100 % epoxy resin	polymerisation for at least 48 h at 60 °C

Supplementary Table 1. Freeze substitution protocol
