# Supplementary material for: Electron tomography of mouse LINC complexes at meiotic telomere attachment sites with and without microtubules
Source: Commun Biol. 2019 Oct 14;2:376. doi: 10.1038/s42003-019-0621-1 (PMC6791847; doi:10.1038/s42003-019-0621-1)
Supplement: Supplementary file 2 — Description of Additional Supplementary Files [file 42003_2019_621_MOESM2_ESM.docx]

**Supplementary Data 1:** Raw/primary data of LINC complex lengths [nm]. Data organized into columns according to the respective tomogram and assigned into two groups with regard to whether or not the LINC complexes at the analyzed attachment sites were close to a microtubule or not.

**Supplementary Movie 1:** Transmission electron tomographic reconstruction and annotation of a meiotic telomere attachment site to the nuclear envelope (NE). The movie sequence shows the progression through the tomogram in z with annotations on individual virtual sections. The combination of virtual sections results in the 3D model of the site. After an initial rotation of the model, the nuclear envelope is faded out to provide better visibility of the filaments. Annotations include the synaptonemal complex (lateral elements in magenta, central element in yellow), attachment plates (lilac) the transverse filaments (blue) and the LINC complexes (light cyan).

A high-resolution version of this movie is available at http://doi.org/10.5281/zenodo.3375530.

**Supplementary Movie 2:** Transmission electron tomographic reconstruction and annotation of a meiotic telomere attachment site to the nuclear envelope (NE) with a microtubule. Movie sequence progresses through the reconstructed tomogram, showing how the annotation of the structures of interest on individual virtual sections combine into a 3D model of the attachment site. The attachment site is close to a microtubule running parallel to the nuclear envelope. Annotations in accordance with Supplementary Movie 1, microtubule annotated in purple.

A high-resolution version of this movie is available at http://doi.org/10.5281/zenodo.3375530.

**Supplementary Data 2:** Underlying data for figure 5.

**Supplementary Data 3:** Underlying data for figure 6.

**Supplementary Data 4:** Underlying data for figure 7.

**Supplementary Data 5:** Underlying data for figure 8.
