# Supplementary material for: Electron tomography of mouse LINC complexes at meiotic telomere attachment sites with and without microtubules
Source: Commun Biol. 2019 Oct 14;2:376. doi: 10.1038/s42003-019-0621-1 (PMC6791847; doi:10.1038/s42003-019-0621-1)
Supplement: Supplementary file 8 — Reporting Summary [file 42003_2019_621_MOESM8_ESM.pdf]

nature research

Corresponding author(s): Prof. Ricardo Benavente

Last updated by author(s): 20.08.2019

Reporting Summary

Nature Research wishes to improve the reproducibility of the work that we publish. This form provides structure for consistency and transparency in reporting. For further information on Nature Research policies, see Authors & Referees and the Editorial Policy Checklist .

Statistics

For all statistical analyses, confirm that the following items are present in the figure legend, table legend, main text, or Methods section.

n/a	Confirmed	
☐	☒	The exact sample size (*n*) for each experimental group/condition, given as a discrete number and unit of measurement
☐	☒	A statement on whether measurements were taken from distinct samples or whether the same sample was measured repeatedly
☐	☒	The statistical test(s) used AND whether they are one- or two-sided<br>*Only common tests should be described solely by name; describe more complex techniques in the Methods section.*
☒	☐	A description of all covariates tested
☒	☐	A description of any assumptions or corrections, such as tests of normality and adjustment for multiple comparisons
☐	☒	A full description of the statistical parameters including central tendency (e.g. means) or other basic estimates (e.g. regression coefficient) AND variation (e.g. standard deviation) or associated estimates of uncertainty (e.g. confidence intervals)
☐	☒	For null hypothesis testing, the test statistic (e.g. *F*, *t*, *r*) with confidence intervals, effect sizes, degrees of freedom and *P* value noted<br>*Give P values as exact values whenever suitable.*
☒	☐	For Bayesian analysis, information on the choice of priors and Markov chain Monte Carlo settings
☒	☐	For hierarchical and complex designs, identification of the appropriate level for tests and full reporting of outcomes
☒	☐	Estimates of effect sizes (e.g. Cohen's *d*, Pearson's *r*), indicating how they were calculated

Our web collection on statistics for biologists contains articles on many of the points above.

Software and code

Policy information about availability of computer code

Data collection	CLC main workbench 7.6.4<br>Zeiss Zen 2 software 10.0 black edition<br>SerialEM
Data analysis	ImageJ 1.52e (visualization)<br>IMOD Version 4.8.62<br>Matlab R2017a (custom scripts)<br>GIMP 2.8 (visualization)<br>Inkscape 0.92 (visualization)

For manuscripts utilizing custom algorithms or software that are central to the research but not yet described in published literature, software must be made available to editors/reviewers. We strongly encourage code deposition in a community repository (e.g. GitHub). See the Nature Research guidelines for submitting code & software for further information.

Data

Policy information about availability of data

All manuscripts must include a data availability statement. This statement should provide the following information, where applicable:

- Accession codes, unique identifiers, or web links for publicly available datasets
- A list of figures that have associated raw data
- A description of any restrictions on data availability

The data that support the findings of this study are available from the corresponding author upon reasonable request.

Field-specific reporting

Please select the one below that is the best fit for your research. If you are not sure, read the appropriate sections before making your selection.

[x] Life sciences [ ] Behavioural & social sciences [ ] Ecological, evolutionary & environmental sciences

For a reference copy of the document with all sections, see nature.com/documents/nr-reporting-summary-flat.pdf

Life sciences study design

All studies must disclose on these points even when the disclosure is negative.

Sample size	Due to homogenicity between samples, the sample size of the two subgroups (with/without microtubules) is sufficient with 11 analyzed tomograms in total. A similar number of tomograms is used in other tomographic studies.
Data exclusions	No data was excluded.
Replication	Filaments were repeatedly verified as LINC complexes using respective antibodies against either SUN1 or KASH5 on frozen tissues of different wildtype C57BL/6J animals. Tomogram acquisition as well as the results of the data analysis between individual tomograms was reproducible.
Randomization	Not relevant to the study.
Blinding	Blinding is not relevant to the study.

Reporting for specific materials, systems and methods

We require information from authors about some types of materials, experimental systems and methods used in many studies. Here, indicate whether each material, system or method listed is relevant to your study. If you are not sure if a list item applies to your research, read the appropriate section before selecting a response.

Materials & experimental systems

n/a	Involved in the study
[ ]	[x] Antibodies
[x]	[ ] Eukaryotic cell lines
[x]	[ ] Palaeontology
[ ]	[x] Animals and other organisms
[x]	[ ] Human research participants
[x]	[ ] Clinical data

Methods

n/a	Involved in the study
[x]	[ ] ChIP-seq
[x]	[ ] Flow cytometry
[x]	[ ] MRI-based neuroimaging

Antibodies

Antibodies used	Primary antibodies used in this study were as follows: rabbit against aa 71-83 and aa 89-104 N-terminus of mouse KASH5 (BIOTEM, Apprieu, France, GenBank accession number NP_958762.2), guinea pig against SUN1 (aa 427-722; Göb et al. 2010), mouse against mouse SYCP3, Abcam; ab97672).
Validation	The antibodies were validated in several single, double and triple indirect immunofluorescence and immunogold assays. The antibodies are specific for murine LINC complex components SUN1 and KASH5 at meiotic telomere attachment sites.

Animals and other organisms

Policy information about studies involving animals; ARRIVE guidelines recommended for reporting animal research

Laboratory animals	C57BL/6J wildtype male mice between the ages of 14 days and 12 weeks.
Wild animals	The study did not involve wild animals.
Field-collected samples	The study did not involve field collected samples.
Ethics oversight	Animal care and experiments were conducted in accordance with the guidelines provided by the German Animal Welfare Act (German Ministry of Agriculture, Health and Economic Cooperation). Animal housing and breeding was approved by the regulatory agency of the city of Würzburg (Reference ABD/OA/Tr; according to §11/1 No. 1 of the German Animal Welfare Act).

Note that full information on the approval of the study protocol must also be provided in the manuscript.
